# On-Ground Processing of Yaogan-24 Remote Sensing Satellite Attitude Data and Verification Using Geometric Field Calibration

**DOI:** 10.3390/s16081203

**Published:** 2016-07-30

**Authors:** Mi Wang, Chengcheng Fan, Bo Yang, Shuying Jin, Jun Pan

**Affiliations:** 1 State Key Laboratory of Information Engineering in Surveying, Mapping and Remote Sensing, Wuhan University, 129 Luoyu Road, Wuhan 430079, China; wangmi@whu.edu.cn (M.W.); jsy@whu.edu.cn (S.J.); panjun1215@whu.edu.cn (J.P.); 2 Computer School, Wuhan University, 129 Luoyu Road, Wuhan 430079, China; 2009106190044@whu.edu.cn; 3 Collaborative Innovation Center of Geospatial Technology, Wuhan University, 129 Luoyu Road, Wuhan 430079, China

**Keywords:** precise attitude estimation, star sensor, gyroscope, bidirectional filter, Yaogan-24 remote sensing satellite, earth observation camera

## Abstract

Satellite attitude accuracy is an important factor affecting the geometric processing accuracy of high-resolution optical satellite imagery. To address the problem whereby the accuracy of the Yaogan-24 remote sensing satellite’s on-board attitude data processing is not high enough and thus cannot meet its image geometry processing requirements, we developed an approach involving on-ground attitude data processing and digital orthophoto (DOM) and the digital elevation model (DEM) verification of a geometric calibration field. The approach focuses on three modules: on-ground processing based on bidirectional filter, overall weighted smoothing and fitting, and evaluation in the geometric calibration field. Our experimental results demonstrate that the proposed on-ground processing method is both robust and feasible, which ensures the reliability of the observation data quality, convergence and stability of the parameter estimation model. In addition, both the Euler angle and quaternion could be used to build a mathematical fitting model, while the orthogonal polynomial fitting model is more suitable for modeling the attitude parameter. Furthermore, compared to the image geometric processing results based on on-board attitude data, the image uncontrolled and relative geometric positioning result accuracy can be increased by about 50%.

## 1. Introduction

Since 2010, China has launched a series of high-resolution optical satellites. On 9 January 2012, China launched the first high-accuracy civil stereo-mapping optical satellite ZY-3 from the Taiyuan satellite launch center. Like Japan’s Advanced Land-Observing Satellite (ALOS) [1], ZY-3 was equipped with three panchromatic (0.50–0.80 μm) time-delay CCD-integrated cameras (nadir, forward, and backward) capable of taking in-track triplet stereo images. Their spatial resolutions were 2.1 m (for the nadir camera) and 3.5 m (for the forward and backward cameras) [2]. On 20 November 2014, the Yaogan-24 remote sensing satellite, with panchromatic spatial resolution within 1 m, was successfully launched. Optical satellite ZY-3-02 will be launched in early 2016, which is similar to ZY-3 satellite. In general, the orbital heights of these optical satellites are 500–800 km, from which a 1 arcsec attitude error could cause 3–5 m ground positioning error if other sources of error are ignored. Therefore, the impact of the accuracy of the satellite’s attitude data on the high-resolution image geometry process could be very significant and is summed up by the statement “one false step will make a great difference”. Thanks to technological advancements in precise orbit determination, microsecond time synchronization, and high-accuracy calibration of internal camera parameters, satellite attitude precision is becoming an important factor restricting the geometric positioning precision of high-resolution optical images, which remains unsolved [3,4,5,6].

To achieve high-precision image geometry processing for attitude determination of a high-resolution optical satellite, many attempts have been made in remote sensing and geoscience applications, including deterministic method such as TRIAD, QUEST, FOAM, SVD, Euler-q, ESOQ-2, which are based on vector observations, and the state filtering estimation method such as SOAR, q-EKF, Filter QUEST, and REQUEST methods [7,8,9,10,11,12]. Meanwhile, correlation algorithms for precision geometry processing of optical images have matured, including (to name a few), the tight geometric-imaging, rational polynomial-fitting, and pixel-pointing angle models, among others [2,3,4,13,14,15,16]. In particular, in recent years platform jittering and sub-meter image processes have become hot topics. Typical methods include tremor detection that are based on the calibration field, and tremor image geometry compensation based on a high-frequency angular displacement sensor [1,17,18,19]. However, to our best knowledge current studies are scarce; most are based mainly on simulation data, but lack the validation of real data. Due to the intrinsic nature of attitude, the issue of how to evaluate and verify its accuracy effectively also remains unsolved. Fortunately, our research has been dedicated to undertake intensive research in this area. Therefore, we introduce what we have undertaken recently to fill the perceived research gap.

Satellite attitude determination accuracy depends not only on the attitude sensor measurement accuracy, but also on the attitude data processing method used [20]. Generally, optical satellite images always uses on-board processing attitude data for geometric processing, which is also used for the satellite attitude control systems. Because the satellite’s attitude control system depends not on the accuracy, but on the robustness of attitude data, onboard processing usually uses a real-time unidirectional Kalman filter for attitude data processing, which is due to the use of past observation data; it relies more on gyro observation information, which will affect the accuracy of attitude for the existing of gyro bias and other error sources, is unable to make full use of the original observation data of attitude sensors to achieve high-precision processing [21]. Therefore, the attitude accuracy of the attitude control system cannot meet the requirements of optical image geometry processing.

To address these problems, the Yaogan-24 remote sensing satellite was taken as an example for doing research and experimental analysis. We will use the bidirectional Kalman filter and overall weighted smoothing method for attitude data processing to enhance the accuracy, a method that can realize high-precision ground processing for attitude data. Meanwhile, we will use the real panchromatic image and digital orthophoto (DOM) and digital elevation (DEM) (DOM/DEM) model of the geometric calibration field to achieve automatic measurements of high-precision control points and high-precision attitude inversions based on the image-intensive matching method. With this approach we can more reasonably evaluate the absolute and relative attitude accuracies.

The remainder of the paper is organized as follows: Section 2 introduces attitude data processing, model construction, and the verification methods. Section 3 presents the collection and analysis of experimental data. Section 4 summarizes our work and presents the study’s conclusions and research perspectives.

## 2. On-Ground Processing for Attitude Data and Verification in a Geometric Calibration Field

### 2.1. The On-Ground Processing Workflow

The Yaogan-24 remote sensing satellite is a high-resolution mapping satellite injected in a Sun-synchronous orbit at an altitude of 645 km. To extend the viewing range of the camera, HRO can swing laterally up to a maximum of 32°. The photographing system consists of a panchromatic linear mapping camera and a multi-spectral camera with a pixel resolution of 1.0 m and 2 m, respectively.

The systems of the satellite attitude sensor for configuration include two German Astro10 star sensors, an APS-made star sensor, four gyro components, a digital Sun sensor, and an infrared Earth sensor. Table 1 shows the details of the Yaogan-24 remote sensing satellite’s star sensor and gyro performance parameters. In addition, the Astro10 star sensor on the satellite was superior to the domestic APS star sensor that was used as an alternative.

Precise attitude data are a precondition for achieving high-precision geometric positioning of high-resolution optical image processing. The goal of attitude determination is to calculate the attitude parameters in the reference coordinate system on the basis of measurements from the attitude sensors. An attitude determination system consists of the attitude sensor and its attitude-determination algorithm [20]. Therefore, attitude accuracy depends on the accuracies of both the measurement and the calibration algorithm. On-satellite attitude sensors on high-resolution optical satellites include an infrared Earth sensor, a Sun sensor, star sensor, and gyro inertial sensors, etc. In general, the data of the star sensor and gyroscope are combined to determine the precise attitude parameters for high-resolution optical satellites [22,23,24].

**Figure 1:**
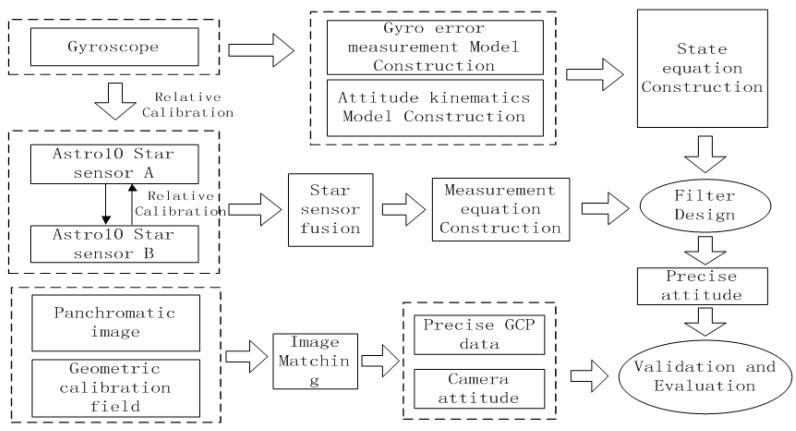
Flow chart for attitude data processing and verification.

Figure 1 shows the on-ground processing workflow for attitude data of the Yaogan-24 remote sensing satellite and its verification. Firstly, we do preprocessing of attitude observation data. Secondly, we construct the measurement model and state model, use the bidirectional Kalman filter and overall weighted smoothing method to realize the optimal satellite attitude estimation. Finally, we use the panchromatic image and reference data of geometric field to verify the relative and absolute accuracy of the estimated attitude data. The workflow includes four major steps:

(1) Measurement equation construction

A star sensor optical axis vector can achieve high precision for positioning a space object, from which an equation of measurements is built. To ensure the quality and reliability of the observational attitude data, we will control the quality of the observational data based on optical axis angle stability.

(2) State equation construction

Gyro data reflect the change in attitude and are used to construct the equation of state on the basis of attitude kinematic equations.

(3) Filtering for information fusion

To estimate the optimal attitude parameters, we use a bidirectional Kalman filter to process attitude data, which are then smoothed overall according to the error covariance matrix.

(4) Control point measurement and attitude precision inversion

To verify the effectiveness of our method, we applied real image and DOM/DEM data in the geometric calibration field for geometric processing and attitude precision inversion.

Before building the state and measurement equations, we must first determine the state variables. The selection of state variables can directly affect the non-linear dimensions of the state and measurement equations. To reduce the matrix order and streamline the attitude determination algorithms, we chose three parameter variables of error quaternion vector, and gyro bias error, as the state variables of the system, that is, $X={\left[\Delta q_{13}{}^{T},\Delta b^{T}\right]}^{T}$, $\Delta q_{13}={\left[\Delta q_{1}\;\;\;\Delta q_{2}\;\;\;\Delta q_{3}\right]}^{T}$.

### 2.2. Measurement Equation Construction

The measurement accuracy of a star sensor’s optical axis is the highest among the three axes, and we will construct the measurement equation based on the optical axis vector. Construction of the star sensor measurement equation includes the following three aspects:

(1) Quality control of observational data from the star sensor

Naturally, certain unusual errors may occur during star sensor observation. Therefore, the quality of the observational data should be controlled first. Suppose that at time t, the quaternion observation value of Sensor A is $q_{t}^{A}={\left[\begin{array}{cccc} q_{0A} & q_{1A} & q_{2A} & q_{3A} \end{array}\right]}^{T}$, and that of Sensor B is $q_{t}^{B}={\left[\begin{array}{cccc} q_{0B} & q_{1B} & q_{2B} & q_{3B} \end{array}\right]}^{T}$. We could place the satellite body into the inertial rotation matrices $R_{A}^{I}$ and $R_{B}^{I}$:

(1) $$R_{A}^{I}=\left[\begin{array}{ccc} {q_{1A}}^{2}-{q_{2A}}^{2}-{q_{3A}}^{2}+{q_{0A}}^{2} & 2(q_{1A}q_{2A}-q_{3A}q_{0A}) & 2(q_{1A}q_{3A}+q_{2A}q_{0A})\\ 2(q_{1A}q_{2A}+q_{3A}q_{0A}) & -{q_{1A}}^{2}+{q_{2A}}^{2}-{q_{3A}}^{2}+{q_{0A}}^{2} & 2(q_{2A}q_{3A}-q_{1A}q_{0A})\\ 2(q_{1A}q_{3A}-q_{2A}q_{0A}) & 2(q_{2A}q_{3A}+q_{1A}q_{0A}) & -{q_{1A}}^{2}-{q_{2A}}^{2}+{q_{3A}}^{2}+{q_{0A}}^{2} \end{array}\right]$$

(2) $$R_{B}^{I}=\left[\begin{array}{ccc} {q_{1B}}^{2}-{q_{2B}}^{2}-{q_{3B}}^{2}+{q_{0B}}^{2} & 2(q_{1B}q_{2B}-q_{3B}q_{0B}) & 2(q_{1B}q_{3B}+q_{2B}q_{0B})\\ 2(q_{1B}q_{2B}+q_{3B}q_{0B}) & -{q_{1B}}^{2}+{q_{2B}}^{2}-{q_{3B}}^{2}+{q_{0B}}^{2} & 2(q_{2B}q_{3B}-q_{1B}q_{0B})\\ 2(q_{1B}q_{3B}-q_{2B}q_{0B}) & 2(q_{2B}q_{3B}+q_{1B}q_{0B}) & -{q_{1B}}^{2}-{q_{2B}}^{2}+{q_{3B}}^{2}+{q_{0B}}^{2} \end{array}\right]$$

Next, we can obtain the optical axis vectors of the two sensors in the inertial reference frame as follows:

(3) $$\begin{array}{l} Z^{A}={\left[\begin{array}{ccc} 2(q_{1A}q_{3A}+q_{2A}q_{0A}) & 2(q_{2A}q_{3A}-q_{1A}q_{0A}) & -{q_{1A}}^{2}-{q_{2A}}^{2}+{q_{3A}}^{2}+{q_{0A}}^{2} \end{array}\right]}^{T}\\ Z^{B}={\left[\begin{array}{ccc} 2(q_{1B}q_{3B}+q_{2B}q_{0B}) & 2(q_{2B}q_{3B}-q_{1B}q_{0B}) & -{q_{1B}}^{2}-{q_{2B}}^{2}+{q_{3B}}^{2}+{q_{0B}}^{2} \end{array}\right]}^{T} \end{array}$$

Next, we could calculate the angle between the two at time *t*:

(4) $$\alpha_{t}=\arccos(Z^{A}\cdot Z^{B})$$

As a rigid body bracket and temperature control device are used among the star sensors, and the axis angle change among the sensors is very small, we are able to control the quality of observational data in the following model:

(5) $$\begin{array}{l} \delta_{m}=\sqrt{\frac{\sum_{i=1}^{N}{\left(\alpha_{i}-\alpha_{cal}\right)}^{2}}{N}}\\ \left\{ \begin{array}{l} \left|\alpha_{i}-\alpha_{cal}\right|\leq \gamma \delta_{m}\;\;\text{normal observation}\\ \left|\alpha_{i}-\alpha_{cal}\right|>\gamma \delta_{m}\;\;\text{abnormal observation} \end{array}\right. \end{array}$$

where $\alpha_{cal}$ represents the angle between the optical axis calibrated in the laboratory, γ represents the threshold value, the general range of 1–3, and $\delta_{m}$ represents the mean square error of the optical axis angle.

(2) Data fusion for multiple star sensors

Data fusion for multiple star sensors uses origin attitude observations, by which the high-precision attitude of the body coordinate relative to the inertial coordinate system can be determined, and the attitude accuracy depends on the optical axis pointing accuracy of the star sensor [25,26]. This operation is a prerequisite for the combination of star sensor and gyro. Assuming that the axis of multiple star sensors in the inertial coordinate is $V_{1CIS}$, $V_{2CIS}$, … $V_{nCIS}$, the body coordinate of the axis is $V_{1Body}$, $V_{2Body}$, … $V_{nBody}$, and the star sensor observation equation would be:

(6) $$V_{iCIS}+v_{3\times 1}={\hat{R}}_{B}^{I}\cdot V_{iBody}\;\;i=1,2,\cdots ,n$$

in which ${\hat{R}}_{B}^{I}$ is the rotation matrix from the body coordinate system to the inertial coordinate system, and $v_{3\times 1}$ is the star sensitive observation error.

We could establish an indirect adjustment model using quaternions as independent variables, which is expressed as:

(7) $$\begin{array}{l} V_{iCIS}+v_{3\times 1}={\hat{R}}_{B}^{I}\cdot V_{iBody}\;\;(i=1,2,\cdots ,n)\\ {{\hat{q}}_{0}}^{2}+{{\hat{q}}_{1}}^{2}+{{\hat{q}}_{2}}^{2}+{{\hat{q}}_{3}}^{2}=1 \end{array}$$

We do a Taylor series expansion on the first item:

(8) $$\begin{array}{cc} v_{3\times 1} & =R_{B}^{I}\cdot V_{iBody}-V_{iCIS}\\ & =R_{B}^{I}(Q_{0})\cdot V_{iBody}+(\frac{\partial R_{B}^{I}}{\partial q_{0}}V_{iBody}dq_{0}+\frac{\partial R_{B}^{I}}{\partial q_{1}}V_{iBody}dq_{1}+\frac{\partial R_{B}^{I}}{\partial q_{2}}V_{iBody}dq_{2}+\frac{\partial R_{B}^{I}}{\partial q_{3}}V_{iBody}dq_{3})-V_{iCIS}\\ {q_{00}}^{2} & +{q_{01}}^{2}+{q_{02}}^{2}+{q_{03}}^{2}+2q_{00}dq_{0}+2q_{01}dq_{1}+2q_{02}dq_{2}+2q_{03}dq_{3}-1=0 \end{array}$$

where $Q_{0}={\left[\begin{array}{cccc} q_{00} & q_{01} & q_{02} & q_{03} \end{array}\right]}^{T}$ is the initial value of the unknown quaternion. The above equation is linearized with restriction of error equations, and could be written in matrix form:

(9) $$\begin{array}{l} V=AX-L\\ CX+W=0 \end{array}$$

Furthermore, we could solve the following equations and the specific derivation process is as follows:

(10) $$\{\begin{array}{c} V=AX-L\\ CX+W=0\\ A^{T}PV+C^{T}K=0 \end{array}$$

(11) $$\begin{array}{l} A^{T}PAX+C^{T}K-A^{T}PL=0\\ K={\left(C{\left(A^{T}PA\right)}^{-1}C^{T}\right)}^{-1}(C{\left(A^{T}PA\right)}^{-1}A^{T}PL+W)\\ X={\left(A^{T}PA\right)}^{-1}(A^{T}PL-C^{T}{\left(C{\left(A^{T}PA\right)}^{-1}C^{T}\right)}^{-1}(C{\left(A^{T}PA\right)}^{-1}A^{T}PL+W)) \end{array}$$

We then calculate the quaternion update:

(12) $$\begin{array}{l} q_{0}=q_{00}+dq_{0}\\ q_{1}=q_{01}+dq_{1}\\ q_{2}=q_{02}+dq_{2}\\ q_{3}=q_{03}+dq_{3} \end{array}$$

We do iterative calculations until $X={\left[\begin{array}{cccc} dq_{0} & dq_{1} & dq_{2} & dq_{3} \end{array}\right]}^{T}$ stabilizes. Therefore, we could calculate the quaternion estimation based on the above model.

(3) Constructing the measurement equation

From the fusion results of the multiple-star sensor observations shown above, we can construct attitude measurement equation. The attitudes of the three axes vectors in the body coordinate system, the measurement values in the inertial coordinate system and the real values are:

(13) $$\left\{ \begin{array}{l} l_{b}^{1}={\left[x_{b}^{1},y_{b}^{1},z_{b}^{1}\right]}^{T}\\ l_{b}^{2}={\left[x_{b}^{2},y_{b}^{2},z_{b}^{2}\right]}^{T}\\ l_{b}^{3}={\left[x_{b}^{3},y_{b}^{3},z_{b}^{3}\right]}^{T} \end{array}\right.,\left\{ \begin{array}{l} l_{mi}^{1}={\left[x_{mi}^{1},y_{mi}^{1},z_{mi}^{1}\right]}^{T}\\ l_{mi}^{2}={\left[x_{mi}^{2},y_{mi}^{2},z_{mi}^{2}\right]}^{T}\\ l_{mi}^{3}={\left[x_{mi}^{3},y_{mi}^{3},z_{mi}^{3}\right]}^{T} \end{array}\right.,\left\{ \begin{array}{l} l_{i}^{1}={\left[x_{i}^{1},y_{i}^{1},z_{i}^{1}\right]}^{T}\\ l_{i}^{2}={\left[x_{i}^{2},y_{i}^{2},z_{i}^{2}\right]}^{T}\\ l_{i}^{3}={\left[x_{i}^{3},y_{i}^{3},z_{i}^{3}\right]}^{T} \end{array}\right.$$

We simplify the measurement equations of:

(14) $$\begin{array}{l} Z(t)=h(X,t)+V(t)\\ h(X,t)={\left[\begin{array}{l} l_{i}^{1}\\ l_{i}^{2}\\ l_{i}^{3} \end{array}\right]}_{9\times 1}=\left[\begin{array}{l} h_{1}\\ h_{2}\\ h_{3} \end{array}\right],V(t)={\left[\begin{array}{l} v_{1}\\ v_{2}\\ v_{3} \end{array}\right]}_{9\times 1} \end{array}$$

As the measurement Equation (14) is continuous and $\hat{X}(t)$ is non-linear, to use the attitude estimation filtering algorithm we first need to focus on the best estimates of $\hat{X}(t)$ to linearize it with the sampling period T, and then do discrete and recursive calculations. The detailed derivation process is as follows:

(15) $$\begin{array}{l} h_{1}=l_{i}^{1}=A_{bi}^{T}(q)l_{b}^{1}\\ A_{bi}(q)=A_{bi}(\Delta q)A_{bi}(\hat{q}) \end{array}$$

where $A_{bi}(q)$ represents the rotational transformation matrix from the inertial to the body coordinate system. As the error quaternion is small, it can be reduced to:

(16) $$\begin{array}{l} A(\Delta q)=\left[\begin{array}{ccc} 1 & 2\Delta q_{3} & -2\Delta q_{2}\\ -2\Delta q_{3} & 1 & 2\Delta q_{1}\\ 2\Delta q_{2} & -2\Delta q_{1} & 1 \end{array}\right]=I_{3\times 3}-2[\Delta \vec{q}\times ]\\ A_{bi}(q)=A_{bi}(\Delta q)A_{bi}(\hat{q})=\left\{I_{3\times 3}-2[\Delta \vec{q}\times ]\right\}A_{bi}(\hat{q}) \end{array}$$

Further solving it:

(17) $$\begin{array}{l} h_{1}=l_{i}^{1}=A_{bi}^{T}(q)l_{b}^{1}\\ =A_{bi}^{T}(\hat{q})l_{b}^{1}-{\left\{2[\Delta \vec{q}\times ]A_{bi}(\hat{q})\right\}}^{T}l_{b}^{1}\\ =A_{bi}^{T}(\hat{q})l_{b}^{1}+2A_{bi}^{T}(\hat{q})[\Delta \vec{q}\times ]l_{b}^{1}\\ =A_{bi}^{T}(\hat{q})l_{b}^{1}-2A_{bi}^{T}(\hat{q})[l_{b}^{1}\times ]\Delta \vec{q} \end{array}$$

We could similarly get:

(18) $$\begin{array}{l} h_{2}=l_{i}^{2}=A_{bi}^{T}(q)l_{b}^{2}\\ =A_{bi}^{T}(\hat{q})l_{b}^{2}-{\left\{2[\Delta \vec{q}\times ]A_{bi}(\hat{q})\right\}}^{T}l_{b}^{2}\\ =A_{bi}^{T}(\hat{q})l_{b}^{2}+2A_{bi}^{T}(\hat{q})[\Delta \vec{q}\times ]l_{b}^{2}\\ =A_{bi}^{T}(\hat{q})l_{b}^{2}-2A_{bi}^{T}(\hat{q})[l_{b}^{2}\times ]\Delta \vec{q} \end{array},\;\;\begin{array}{l} h_{3}=l_{i}^{3}=A_{bi}^{T}(q)l_{b}^{3}\\ \;=A_{bi}^{T}(\hat{q})l_{b}^{3}-{\left\{2[\Delta \vec{q}\times ]A_{bi}(\hat{q})\right\}}^{T}l_{b}^{3}\\ \;=A_{bi}^{T}(\hat{q})l_{b}^{3}+2A_{bi}^{T}(\hat{q})[\Delta \vec{q}\times ]l_{b}^{3}\\ \;=A_{bi}^{T}(\hat{q})l_{b}^{3}-2A_{bi}^{T}(\hat{q})[l_{b}^{3}\times ]\Delta \vec{q} \end{array}$$

Now we calculate the measurement matrix:

(19) $$\frac{\partial h_{1}}{\partial \Delta \vec{q}}=-2A_{bi}^{T}(\hat{q})[l_{b}^{1}\times ],\;\frac{\partial h_{2}}{\partial \Delta \vec{q}}=-2A_{bi}^{T}(\hat{q})[l_{b}^{2}\times ],\;\;\;\frac{\partial h_{3}}{\partial \Delta \vec{q}}=-2A_{bi}^{T}(\hat{q})[l_{b}^{3}\times ]$$

Therefore, Equation (14) becomes linear and discrete:

(20) $$\begin{array}{l} Z_{k}=H_{k}X_{k}+V_{k}\\ H_{k}={\frac{\partial h[X(t_{k}),t_{k}]}{\partial X(t_{k})}|}_{X(t_{k})={\hat{X}}_{k/k-1}}={\left[\begin{array}{ll} \frac{\partial h_{1}}{\partial \Delta \vec{q}} & 0_{3\times 3}\\ \frac{\partial h_{2}}{\partial \Delta \vec{q}} & 0_{3\times 3}\\ \frac{\partial h_{3}}{\partial \Delta \vec{q}} & 0_{3\times 3} \end{array}\right]}_{9\times 9}={\left[\begin{array}{ll} -2A_{bi}^{T}(\hat{q})[l_{b}^{1}\times ] & 0_{3\times 3}\\ -2A_{bi}^{T}(\hat{q})[l_{b}^{2}\times ] & 0_{3\times 3}\\ -2A_{bi}^{T}(\hat{q})[l_{b}^{3}\times ] & 0_{3\times 3} \end{array}\right]}_{9\times 9}\\ Z_{k}={\left[\begin{array}{l} l_{mi}^{1}-A_{bi}^{T}(\hat{q})l_{b}^{1}\\ l_{mi}^{2}-A_{bi}^{T}(\hat{q})l_{b}^{2}\\ l_{mi}^{3}-A_{bi}^{T}(\hat{q})l_{b}^{3} \end{array}\right]}_{9\times 1} \end{array}$$

$V_{k}$ is observation noise sequence, satisfying:

(21) $$\left\{ \begin{array}{l} E(V_{k})=0\\ E(V_{k}V_{j}^{T})=R_{k}\delta_{kj} \end{array}\right.$$

where δ represents the Dirichlet function and $R_{k}$ is the covariance matrix of the measurement noise. Assuming that the main axis of the observation error is $\sigma_{s}$, we obtain $R_{k}=\sigma_{s}{}^{2}I_{9\times 9}$.

### 2.3. Construction of the State Equation Based on the Gyroscope and Attitude Kinematic Equations

The measurement model of a gyro, a common inertial attitude sensor, is critical in attitude determination. The gyro output angular velocity is used to integrate and preset the next-time satellite attitude, in which gyro bias is treated as an estimated additional state amount; the measurement data of the gyroscope are used directly in the state equation, but are reflected in the measurement equation. According to the configuration of the on-satellite gyroscope feature, a gyroscope measurement model is:

(22) $$\omega_{g}=\omega +b+\eta_{g}$$

in which $\omega_{g}$ represents the gyro output-measured values; ω is the rotation speed of the satellite body coordinate system relative to the inertial space coordinate; b is the gyro bias; $\eta_{g}$ is the gyro measurement noise; $\sigma_{g}$ is the mean square error of gyro measurement noise; and δ represents Dirichlet function, satisfying:

(23) $$\begin{array}{l} E(\eta_{g}(t))=0\\ E(\eta_{g}(t)\eta_{g}^{T}(t^{\prime }))=\sigma_{g}{}^{2}\delta (t-t^{\prime }) \end{array}$$

A gyro bias amount is not static and meets the following random walk model, assuming the gyro bias is driven by white noise, i.e.,:

(24) $$\begin{array}{l} \dot{b}=\eta_{b}\\ \left\{ \begin{array}{l} E(\eta_{b}(t))=0\\ E(\eta_{b}(t)\eta_{b}{}^{T}(t^{\prime }))=\sigma_{b}{}^{2}\delta (t-t^{\prime }) \end{array}\right. \end{array}$$

Furthermore, we assume that the two types of noise are independent. $\eta_{b}$ represents the gyro bias white noise; and $\sigma_{b}$ is the mean square error of the gyro bias white noise.

With the quaternion kinematics:

(25) $$\dot{q}=\frac{1}{2}q⊗\omega_{bi}$$

in which q represents the attitude of the satellite body coordinate system relative to the inertial coordinate system and ${\vec{\omega }}_{bi}$ represents the speed in the body coordinate system of the inertial coordinate system relative to the satellite body, we can obtain an integrator quaternion. As the angle rate of the gyro measurement contains, for example, measurement error and bias error, we can only obtain the corresponding $\hat{q}$ and ${\hat{\omega }}_{bi}$ estimates.

The error between the real satellite attitude quaternion *q* and the quaternion estimates $\hat{q}$ can be expressed as $\Delta q={\left[\Delta q_{0}\;\;\;\Delta q_{1}\;\;\;\Delta q_{2}\;\;\;\Delta q_{3}\right]}^{T}$, and using the error quaternion to represent the error, we have:

(26) $$q=\hat{q}⊗\Delta q$$

where the error quaternion Δq represents a small rotation angle in which $\Delta q_{0}\approx 1$, so we just need to consider the vector part of the quaternion error, and the error quaternion can be reduced into three independent variables.

Change Equation (26) into:

(27) $$\Delta q={\hat{q}}^{-1}⊗q$$

and calculate the derivative of both sides:

(28) $$\Delta \dot{q}={\dot{\hat{q}}}^{-1}⊗q+{\hat{q}}^{-1}⊗\dot{q}$$

Furthermore, from Equation (28):

(29) $$\begin{array}{cc} \Delta \dot{q} & ={\dot{\hat{q}}}^{-1}⊗q+{\hat{q}}^{-1}⊗\dot{q}\\ & =-\frac{1}{2}{\hat{\omega }}_{bi}⊗{\hat{q}}^{-1}⊗q+\frac{1}{2}{\hat{q}}^{-1}⊗q⊗\omega_{bi}\\ & =-\frac{1}{2}{\hat{\omega }}_{bi}⊗\Delta q+\frac{1}{2}\Delta q⊗\omega_{bi} \end{array}$$

from which $\Delta \omega_{bi}=\omega_{bi}-{\hat{\omega }}_{bi}$ is obtained:

(30) $$\Delta \dot{q}=-\frac{1}{2}{\hat{\omega }}_{bi}⊗\Delta q+\frac{1}{2}\Delta q⊗{\hat{\omega }}_{bi}+\frac{1}{2}\Delta q⊗\Delta \omega_{bi}$$

Because the error quaternion is a small amount, we could obtain:

(31) $$\begin{array}{l} \Delta q={\left[\Delta q_{0}\;\;\;\Delta q_{1}\;\;\;\Delta q_{2}\;\;\;\Delta q_{3}\right]}^{T}={\left[1\;\;\;0\;\;\;0\;\;\;0\right]}^{T}\\ \frac{1}{2}\Delta q⊗\Delta \omega_{bi}=\frac{1}{2}\left[\begin{array}{ll} \Delta q_{0} & -\Delta {\vec{q}}^{T}\\ \Delta \vec{q} & \Delta q_{0}I_{3\times 3}-[\Delta \vec{q}\times ] \end{array}\right]\left[\begin{array}{c} 0\\ \Delta {\vec{\omega }}_{bi} \end{array}\right]\\ =\frac{1}{2}\left[\begin{array}{c} 0\\ \Delta {\vec{\omega }}_{bi}+O(\left|\Delta \vec{q}\right|\left|\Delta {\vec{\omega }}_{bi}\right|) \end{array}\right] \end{array}$$

By incorporating Equations (29) and (30) into (31), and ignoring the second-order small quantities, we can derive kinematic equations based on the error quaternion:

(32) $$\begin{array}{l} \Delta \dot{\vec{q}}=\left[\begin{array}{l} \Delta {\dot{q}}_{1}\\ \Delta {\dot{q}}_{2}\\ \Delta {\dot{q}}_{3} \end{array}\right]=-[{\hat{\vec{\omega }}}_{bi}\times ]\Delta \vec{q}-\frac{1}{2}\Delta b-\frac{1}{2}\eta_{g}\\ \Delta {\dot{q}}_{0}=0\\ \Delta \dot{b}=\eta_{b} \end{array}$$

Using Equation (32), we can constitute a linear filtering state equation based on the state $X_{6\times 1}={\left[\Delta {\vec{q}}^{T}\;\;\;\Delta b^{T}\right]}^{T}$, and get:

(33) $$\begin{array}{l} \dot{X}(t)=F(t)X(t)+W(t)\\ F(t)=\left[\begin{array}{cc} -[{\hat{\omega }}_{bi}\times ] & -0.5I_{3\times 3}\\ 0_{3\times 3} & 0_{3\times 3} \end{array}\right]\\ W(t)={\left[-0.5\eta_{g}\;\;\;\eta_{b}\right]}^{T} \end{array}$$

Equation (33) is a continuous dynamic system filter and will be linear and discrete:

(34) $$\begin{array}{l} X_{k}=\Phi_{k/k-1}X_{k-1}+\Gamma_{k-1}W_{k-1}\\ X(t_{k})=\Phi (t_{k},t_{k-1})X(t_{k-1})+\int_{t_{k-1}}^{t_{k}}\Phi (t_{k},\tau )W(\tau )d\tau \end{array}$$

When a sampling interval is small, the calculation of the state transition matrix will be:

(35) $$\begin{array}{l} \dot{\Phi }(t,t_{k-1})=F(t)\Phi (t,t_{k-1})\\ \Phi (t_{k-1},t_{k-1})=I\\ \Phi (t_{k},t_{k-1})=\exp\int_{t_{k-1}}^{t_{k}}F(t)dt \end{array}$$

When the filter period $T(T=t_{k}-t_{k-1})$ is small, F(t) can be approximated into a constant matrix:

(36) $$\begin{array}{l} F(t)\approx F(t_{k-1})\;\;\;t_{k-1}\leq t\leq t_{k}\\ \Phi (t_{k},t_{k-1})=\exp TF(t_{k-1}) \end{array}$$

In addition, the system noise sequence $W_{k-1}$ in the state equation and the driving array $\Gamma_{k-1}$ can be expressed as:

(37) $$\begin{array}{l} \Gamma_{k-1}=\int_{t_{k-1}}^{t_{k}}\Phi (t_{k},\tau )d\tau =T\cdot I\\ W_{k-1}={\left[-0.5\eta_{g}\;\;\;\eta_{b}\right]}^{T}\\ E\left\{W_{k}\right\}=0,E\left\{W_{k},W_{j}{}^{T}\right\}=Q_{k}\delta_{kj}\\ Q_{k}=diag(0.25\sigma_{g}{}^{2}I_{3\times 3}\;\;\;\sigma_{b}{}^{2}I_{3\times 3}) \end{array}$$

### 2.4. Information Fusion Filter Design

**Figure 2:**
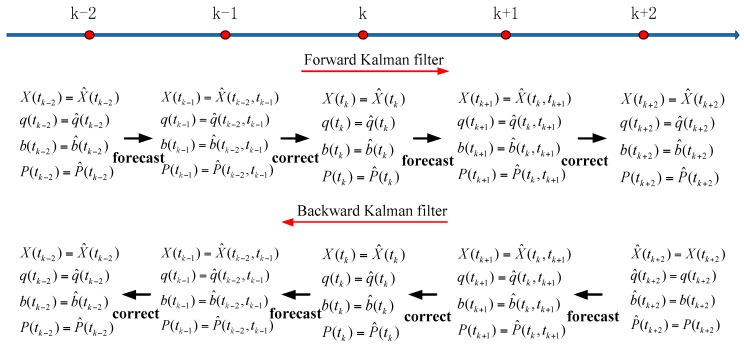
Schematic diagram of attitude data processing with bidirectional Kalman filter.

On the basis of the measurement equation and the state equation, we use the bidirectional Kalman filter and overall weighted smoothing method to realize the optimal satellite attitude estimation and derive it specifically [27,28]. Figure 2 shows the schematic diagram of attitude data processing with the bidirectional Kalman filter.

(1) Attitude forecast processing

When a star sensor does not output a measured value, a gyro measurement model based on the following equation at time $t_{k-1}$ is integrated, from which the satellite quaternion ${\left({\hat{q}}_{bi}\right)}_{k/k-1}$ forecast can be derived:

(38) $$\begin{array}{l} \hat{q}(t_{k/k-1})=e^{\frac{1}{2}\int_{t_{k-1}}^{t_{k}}\Omega ({\hat{\omega }}_{bi}(t_{k-1}))dt}\cdot \hat{q}(t_{k-1})\\ =\left[I+\frac{\frac{1}{2}\Delta \Theta }{1!}+\frac{{\left(\frac{1}{2}\Delta \Theta \right)}^{2}}{2!}+\frac{{\left(\frac{1}{2}\Delta \Theta \right)}^{3}}{3!}+\cdots \right]\cdot \hat{q}(t_{k-1})\\ \Delta \Theta =\int_{t_{k-1}}^{t_{k}}\Omega ({\hat{\omega }}_{bi}(t_{k-1}))dt=\int_{t_{k-1}}^{t_{k}}\left[\begin{array}{cccc} 0 & -\omega_{x} & -\omega_{y} & -\omega_{z}\\ \omega_{x} & 0 & \omega_{z} & -\omega_{y}\\ \omega_{y} & -\omega_{z} & 0 & \omega_{x}\\ \omega_{z} & \omega_{y} & -\omega_{x} & 0 \end{array}\right]dt=\left[\begin{array}{llll} 0 & -\Delta \theta_{x} & -\Delta \theta_{y} & -\Delta \theta_{z}\\ \Delta \theta_{x} & 0 & \Delta \theta_{z} & -\Delta \theta_{y}\\ \Delta \theta_{y} & -\Delta \theta_{z} & 0 & \Delta \theta_{x}\\ \Delta \theta_{z} & \Delta \theta_{y} & -\Delta \theta_{x} & 0 \end{array}\right]\\ \Delta \Theta^{2}=-\Delta \theta^{2}I\\ \Delta \theta^{2}=\Delta \theta_{x}{}^{2}+\Delta \theta_{y}{}^{2}+\Delta \theta_{z}{}^{2} \end{array}$$

where $\Delta \theta_{x},\Delta \theta_{y},\Delta \theta_{z}$ represent the incremented angles of the gyro in the *X*, *Y*, and *Z* axes in a sampling interval $\left[t_{k-1},t_{k}\right]$. Furthermore, the recurrence relations are derived as follows:

(39) $$\begin{array}{l} \hat{q}(t_{k/k-1})=e^{\frac{1}{2}\int_{t_{k-1}}^{t_{k}}\Omega ({\hat{\omega }}_{bi}(t_{k-1}))dt}\cdot \hat{q}(t_{k-1})=\left\{I+I\left[\begin{array}{l} \frac{\frac{\Delta \Theta }{2}}{1!}+\frac{-{\left(\frac{\Delta \theta }{2}\right)}^{2}}{2!}+\frac{-{\left(\frac{\Delta \theta }{2}\right)}^{2}\frac{\Delta \Theta }{2}}{3!}+\frac{{\left(\frac{\Delta \theta }{2}\right)}^{4}}{4!}\\ +\frac{{\left(\frac{\Delta \theta }{2}\right)}^{4}\frac{\Delta \Theta }{2}}{5!}+\frac{-{\left(\frac{\Delta \theta }{2}\right)}^{6}}{6!}+\cdots \end{array}\right]\right\}\cdot \hat{q}(t_{k-1})\\ =\left\{I\left[1-\frac{{\left(\frac{\Delta \theta }{2}\right)}^{2}}{2!}+\frac{{\left(\frac{\Delta \theta }{2}\right)}^{4}}{4!}-\frac{{\left(\frac{\Delta \theta }{2}\right)}^{6}}{6!}+\cdots \right]+\frac{\Delta \Theta }{2}\cdot \frac{2}{\Delta \theta }\left[\frac{\frac{\Delta \theta }{2}}{1!}-\frac{{\left(\frac{\Delta \theta }{2}\right)}^{3}}{3!}+\frac{{\left(\frac{\Delta \theta }{2}\right)}^{5}}{5!}-\cdots \right]\right\}\cdot \hat{q}(t_{k-1})\\ =\left[I\cos\frac{\Delta \theta }{2}+\frac{\Delta \Theta }{\Delta \theta }\sin\frac{\Delta \theta }{2}\right]\cdot \hat{q}(t_{k-1}) \end{array}$$

Therefore, with Equation (39), the satellite quaternion predictive value $\hat{q}(t_{k/k-1})$ can be obtained.

The equation specific for the gyro bias predictive value ${\hat{b}}_{k/k-1}$ is:

(40) $${\hat{b}}_{k/k-1}={\hat{b}}_{k-1}$$

In addition, the equation specific for the predictive value ${\hat{P}}_{k/k-1}$of error covariance matrix is:

(41) $${\hat{P}}_{k/k-1}=\Phi_{k/k-1}{\hat{P}}_{k-1}\Phi_{k/k-1}^{T}+\Gamma_{k-1}Q_{k-1}\Gamma_{k-1}^{T}$$

(2) Attitude correction processing

At time $t_{k}$, the observation matrix $H_{k}$ can be calculated according to the measurement equation, and the filter gain can be calculated using the following equation:

(42) $$K_{k}=P_{k/k-1}H_{k}^{T}{\left[H_{k}P_{k/k-1}H_{k}^{T}+R_{k}\right]}^{-1}$$

Correspondingly, the correction on filter status updates is:

(43) $${\hat{X}}_{k}={\hat{X}}_{k/k-1}+K_{k}(Z_{k}-H_{k}{\hat{X}}_{k/k-1})$$

After obtaining the state variable ${\hat{X}}_{k}={\left[\Delta {\hat{\vec{q}}}_{k}{}^{T}\;\;\;\Delta {\hat{b}}_{k}{}^{T}\right]}^{T}$ at time $t_{k}$, the gyro bias can be corrected by the conventional method:

(44) $${\hat{b}}_{k}={\hat{b}}_{k/k-1}+\Delta {\hat{b}}_{k}$$

The corrected values ${\left({\hat{q}}_{bi}\right)}_{k}$ at time $t_{k}$ are:

(45) $${\left({\hat{q}}_{bi}\right)}_{k}={\left({\hat{q}}_{bi}\right)}_{k/k-1}⊗{\left(\Delta {\hat{q}}_{bi}\right)}_{k}$$

As the constraint on the quaternion modulus equals 1, we can obtain the following results:

(46) $${\left(\Delta {\hat{q}}_{bi}\right)}_{k}=\left[\begin{array}{c} \sqrt{1-\Delta {\hat{\vec{q}}}_{k}{}^{T}\Delta {\hat{\vec{q}}}_{k}}\\ \Delta {\hat{\vec{q}}}_{k} \end{array}\right]$$

The error covariance matrix can then be updated:

(47) $$P_{k}=(I-K_{k}H_{k})P_{k/k-1}{\left(I-K_{k}H_{k}\right)}^{T}+K_{k}R_{k}K_{k}^{T}$$

$H_{k}$ will be determined using the updated quaternion estimation, putting the filtering process in better convergence. After adjustment for the satellite attitude quaternion and gyro bias, the predicted value of the state variable is zero, and the state variable ${\hat{X}}_{k}$ must be reset to zero.

(3) Covariance-weighted smoothing

Results of the optimal estimation are obtained by averaging the forward and backward state estimates with the weights based on their error covariance matrix, which minimizes the covariance of the optimal estimation results. The algorithm for this covariance-weighted smoothing is as follows:

(48) $$\begin{array}{l} {\hat{q}}_{fb}(k)={\hat{q}}_{b}{}^{-1}(k)⊗{\hat{q}}_{f}(k)\\ \Delta {\hat{x}}_{fb}(k)={\left[\begin{array}{cc} \mathrm{sgn}({\hat{q}}_{fb0})[\begin{array}{ccc} {\hat{q}}_{fb1} & {\hat{q}}_{fb2} & {\hat{q}}_{fb3} \end{array}] & {\left({\hat{b}}_{f}(k)-{\hat{b}}_{b}(k)\right)}^{T} \end{array}\right]}^{T}\\ {\hat{P}}_{s}(k)={\left({\hat{P}}_{f}{}^{-1}(k)+{\hat{P}}_{b}{}^{-1}(k)\right)}^{-1}\\ \Delta {\hat{x}}_{s}(k)={\left[\begin{array}{cc} \Delta {\hat{\vec{q}}}_{s}{}^{T}(k) & \Delta {\hat{b}}_{s}{}^{T}(k) \end{array}\right]}^{T}={\hat{P}}_{s}(k){\hat{P}}_{f}{}^{-1}(k)\Delta {\hat{x}}_{fb}(k)\\ \Delta {\hat{q}}_{s0}(k)=sqrt(1-\Delta {\hat{\vec{q}}}_{s1}{}^{T}(k)\Delta {\hat{\vec{q}}}_{s1}{}^{T}(k)-\Delta {\hat{\vec{q}}}_{s2}{}^{T}(k)\Delta {\hat{\vec{q}}}_{s2}{}^{T}(k)-\Delta {\hat{\vec{q}}}_{s3}{}^{T}(k)\Delta {\hat{\vec{q}}}_{s3}{}^{T}(k))\\ {\hat{q}}_{s}={\hat{q}}_{b}(k)⊗\Delta {\hat{q}}_{s}(k)\;\;\;{\hat{b}}_{s}(k)={\hat{b}}_{b}(k)+\Delta {\hat{b}}_{s}(k) \end{array}$$

in which ⊗ represents quaternion multiplication, *f* is the forward filtering result, *b* is the backward one, and *s* is the covariance-weighted smoothing result.

### 2.5. Attitude Data Model Construction

The high-resolution optical satellite equips the line push-broom camera in an imaging frequency up to tens of thoUSAnds of hertz, while the frequency for the attitude data is much smaller. The imaging frequency means number of image lines taken by line push-broom camera per second. Optical image geometric correction requires an accurate attitude for each line; therefore, a reasonable model should be used to meet the image geometry processing requirement. Common fitting methods for the attitude data include [29,30,31]:

(1) Lagrange polynomial interpolation

Lagrange polynomial interpolation is a common interpolation method because it is simple, fast, and widely used. The attitude expression parameter contains Euler angles and quaternions. Assuming that (φ,ω,κ) represents the Euler angle parameter and $\left(q_{0},q_{1},q_{2},q_{3}\right)$ represents quaternion parameters, the specific interpolation model would be:

(49) $$\begin{array}{l} \varphi =\sum_{j=1}^{n}\varphi_{j}W_{j},\omega =\sum_{j=1}^{n}\omega_{j}W_{j},\kappa =\sum_{j=1}^{n}\kappa_{j}W_{j}\\ W_{j}=\prod_{\begin{array}{l} k=1\\ k\neq j \end{array}}^{n}\frac{t-t_{k}}{t_{j}-t_{k}} \end{array}$$

(50) $$\begin{array}{l} q_{1}=\sum_{j=1}^{n}q_{1j}W_{j},q_{2}=\sum_{j=1}^{n}q_{2j}W_{j},q_{3}=\sum_{j=1}^{n}q_{3j}W_{j}\\ q_{0}=\pm \sqrt{\left(1-q_{1}^{2}-q_{2}^{2}-q_{3}^{2}\right)} \end{array}$$

(2) Orthogonal polynomial fitting

Unlike ordinary polynomial fitting models, the orthogonal polynomial model can effectively avoid a pathological matrix. The orthogonal polynomial fitting in the m−1 order of the φ parameter can be expressed as:

(51) $$P_{\varphi }\left(t\right)=a_{0}+a_{1}t+a_{2}t^{2}+\cdots +a_{m-1}t^{m-1},\;(m\leq n)$$

Assuming the above equation is a linear combination of the orthogonal polynomials $\delta_{j}\left(t\right)\left(j=0,1,\cdots ,m-1\right)$, we can obtain:

(52) $$P_{\varphi }\left(t\right)=c_{0}\delta_{0}\left(t\right)+c_{1}\delta_{1}\left(t\right)+\cdots +c_{m-1}\delta_{m-1}\left(t\right)$$

in which the orthogonal polynomials $\delta_{j}\left(t\right)$ can be constructed with a recursive equation as shown below:

(53) $$\delta_{0}(t)=1;\delta_{1}(t)=(t-\alpha_{1});\delta_{j}(t)=(t-\alpha_{j})\delta_{j-1}(t)-\beta_{j}\delta_{j-2}(t),j=2,\cdots ,m-1$$

The orthogonal polynomial fitting principle of Euler angle parameters ω,κ and quaternion are the same as above, so we could get:

(54) $$\begin{array}{l} P_{q_{1}}\left(t\right)=\sum_{k=0}^{m-1}c_{q_{1}k}\delta_{q_{1}k}(t),P_{q_{2}}\left(t\right)=\sum_{k=0}^{m-1}c_{q_{2}k}\delta_{q_{2}k}(t),P_{q_{3}}\left(t\right)=\sum_{k=0}^{m-1}c_{q_{3}k}\delta_{q_{3}k}(t)\\ q_{0}=\pm \sqrt{\left(1-q_{1}^{2}-q_{2}^{2}-q_{3}^{2}\right)} \end{array}$$

(3) Spherical linear interpolation (SLERP) model

If $q_{1},q_{2}$ are considered as two points in four-dimensional space on a unit ball, the SLERP will go along the shortest arc for connection, which can be used in quaternion interpolation at a constant speed. Therefore, we can obtain:

(55) $$\begin{array}{l} q(t)=C_{1}(t)q_{1}+C_{2}(t)q_{2}\\ C_{1}(t)=\frac{\sin(1-t)\theta }{\sin\theta },C_{2}(t)=\frac{\sin t\theta }{\sin\theta }\\ q(t)=\frac{\sin(1-t)\theta }{\sin\theta }q_{1}+\frac{\sin t\theta }{\sin\theta }q_{2}\\ \theta =\cos^{-1}\langle q_{1}\cdot q_{2}\rangle =\cos^{-1}(q_{10}\cdot q_{20}+q_{11}\cdot q_{21}+q_{12}\cdot q_{22}+q_{13}\cdot q_{23}) \end{array}$$

### 2.6. Precise Attitude Inversion in a Geometric Calibration Field

**Figure 3:**
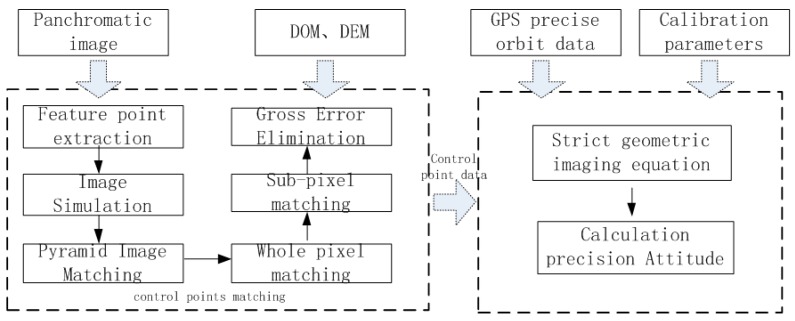
Flowchart of precision control-point matching and high-precision attitude inversion in a geometric calibration field.

Due to the intrinsic nature of attitude, it is difficult to evaluate and verify its accuracy effectively. Based on real panchromatic images and the DOM/DEM of the geometric calibration field, image-dense matching is used to achieve automatic measurement of a high-precision control point, while a strict geometric imaging equation will be used to achieve attitude calculation for earth observation cameras. Furthermore, we could verify and evaluate the accuracy of attitude determination algorithm. Figure 3 displays the flowchart of precision control-point matching and high-precision attitude inversion in a geometric calibration field. It is the core of on-ground processing for attitude data of the Yaogan-24 remote sensing satellite and its verification. It comprises the following key steps of processing that image feature point extraction between the real panchromatic images and the DOM of the geometric calibration field, image simulation, pyramid image matching, whole pixel matching, sub pixel matching and gross error elimination [32,33,34]. The method of above-mentioned could achieve the matching accuracy of sub pixel.

The attitude accuracy includes two aspects: absolute and relative accuracies. Absolute accuracy means the external error including the datum error, while relative accuracy means the internal error after the deduction of datum error. For the first, we will analyze the geometric positioning accuracy of a panchromatic image to evaluate the absolute accuracy; for the second, we will take the inversion attitude of the panchromatic camera as a reference to evaluate the relative accuracy. The absolute and relative accuracy of inversion attitude could respectively reach 0.3 arcsec and 0.06 arcsec under the following conditions:

(a) The Yaogan-24 remote sensing satellite uses dual-frequency GPS observations, precise ephemeris and dynamic model to determine the orbit, by which centimeter-level accuracy can be achieved on the orbit;
(b) Satellite payloads have achieved high-precision time synchronization, and the synchronization error is subtle;
(c) The camera internal parameters have been precisely calibrated in the pixel pointing angle model, and the calibration accuracy is better than 0.3 pixel;
(d) The geometric calibration field is used, in which the absolute and relative accuracy of the control points are respectively within 1 m and 0.2 m in plane.

The camera calibration model used in this paper is [3,4,5]:

(56) $$\begin{array}{l} {\left(V_{\text{Image}}\right)}_{\text{cam}}={\left(\frac{x}{f},\frac{y}{f},1\right)}^{T}={\left(\tan(\psi_{x}(s)),\tan(\psi_{y}(s)),1\right)}^{T}\\ \left\{ \begin{array}{l} \psi_{x}(s)=ax_{0}+ax_{1}\times s+ax_{2}\times s^{2}+ax_{3}\times s^{3}\\ \psi_{y}(s)=ay_{0}+ay_{1}\times s+ay_{2}\times s^{2}+ay_{3}\times s^{3} \end{array}\right. \end{array}$$

In which $\left(\psi_{x}(s),\psi_{y}(s)\right)$ represents the direction angle of probe element *s*; $ax_{0},ax_{1},ax_{2},ax_{3},ay_{0},ay_{1},ay_{2},ay_{3}$ represent the fitting coefficients of pointing angle; x,y represent the coordinate of probe element in camera coordinate system; f represents the focal length of camera; ${\left(V_{\text{Image}}\right)}_{\text{cam}}$ represents the vector of probe element. With Equation (52), we could get that the camera internal error model is simplified, and avoid the coupling of error parameters, when comparing to standard camera intrinsic parameters model.

The tight geometric imaging model used in this paper is:

(57) $$\left(\begin{array}{c} \tan(\psi_{x}(s))\\ \tan(\psi_{y}(s))\\ 1 \end{array}\right)=\lambda R_{\text{body}}^{\text{cam}}\left(R_{\text{J2000}}^{\text{body}}R_{\text{wgs}}^{\text{J2000}}{\left[\begin{array}{l} X_{g}-X_{\text{gps}}\\ Y_{g}-Y_{\text{gps}}\\ Z_{g}-Z_{\text{gps}} \end{array}\right]}_{\text{wgs}}-{\left[\begin{array}{l} B_{X}\\ B_{Y}\\ B_{Z} \end{array}\right]}_{\text{body}}\right)$$

In which $\left(X_{g},Y_{g},Z_{g}\right)$ represent object square coordinates of object points; $\left(X_{\text{gps}},Y_{\text{gps}},Z_{\text{gps}}\right)$ and $\left(B_{X},B_{Y},B_{Z}\right)$ respectively represent the object space coordinates of the camera center and GPS eccentric error; λ represents a scaling factor; $R_{\text{WGS}}^{J2000},R_{J2000}^{\text{body}},$ and $R_{body}^{\text{cam}}$ represent the rotation matrices of, respectively, the WGS84 coordinate system to the J2000 coordinate system, the satellite the J2000 coordinate to body coordinate system, and the satellite body coordinate to the camera coordinate system.

With Equation (57), we could obtain a conversion model between the observation vector in the J2000 coordinate and observation vector in the camera measurement coordinates:

(58) $$\lambda^{-1}{\left(R_{\text{body}}^{\text{cam}}\right)}^{-1}\left(\begin{array}{c} \tan(\psi_{x}(s))\\ \tan(\psi_{y}(s))\\ 1 \end{array}\right)=R_{\text{J2000}}^{\text{body}}R_{\text{wgs}}^{\text{J2000}}{\left[\begin{array}{l} X_{g}-X_{\text{gps}}\\ Y_{g}-Y_{\text{gps}}\\ Z_{g}-Z_{\text{gps}} \end{array}\right]}_{\text{wgs}}-{\left[\begin{array}{l} B_{X}\\ B_{Y}\\ B_{Z} \end{array}\right]}_{\text{body}}$$

With the line push-broom camera, when a non-collinear observation vector on a matching control point in each scan line is ≥2, the attitude parameters along the scan line at certain time can be calculated from Equation (58). In theory, to ensure the precision and reliability of the attitude parameters, a larger number of matching control points are required and they are distributed evenly along the scan line [35,36]. The attitude accuracy depends mainly on the accuracy of the GPS orbit accuracy, DEM/DOM accuracy of the reference calibration field data, and the number and distribution of the control points per scan line [34,37,38,39]. The frequency of the inversion attitude should be equal to that of the linear array camera imaging, and can reach tens of thousands of hertz. However, changes in the calibration field are relatively large, making it impossible to match the control points of each line. Therefore, we will down-sample the frequency to tens of hertz.

## 3. Experiment and Discussion

An experiment was carried out using the data provided by the Yaogan-24 remote sensing satellite that was launched on 20 November 2014. The on-ground attitude data processing algorithm has been applied in the ground processing system in the China Resources Satellite Application Center.

### 3.1. Experimental Data

(1) Observation data of the Yaogan-24 remote sensing satellite

The Yaogan-24 remote sensing satellite’s original observation data used for experimental analysis mainly includes dual-frequency GPS original observations, original observations of Astro10 A and B, gyro observation data, line time data of imaging, and panchromatic image data. The data mentioned above were acquired during the satellite in orbit test.

(2) Geometric calibration field

**Figure 4:**
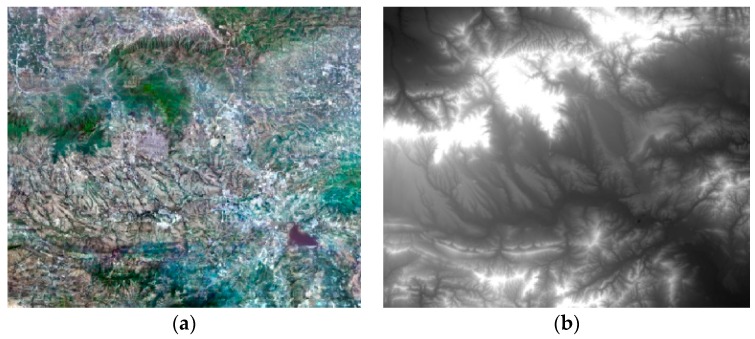
Reference data of the Songshan calibration field. (**a**) Digital orthophoto; (**b**) Digital elevation model.

The geometric calibration fields included Songshan, Anyang, Dongying, Sanya, Taiyuan, and Yili, all in China. In this paper, the Songshan and Anyang calibration fields were used. The Songshan field is located in Henan Province, central China, and features a hilly terrain, 112°42′–113°54′ E/34°13′–35°2′ N, coverage 100 × 80 = 8000 km^2^, average altitude of approximately 500 m (the highest point is at 1491.73 m), and a maximum fluctuation ≤2000 m. The Songshan calibration field provides a region of 1:2000-scale digital orthophoto (DOM) and digital elevation model (DEM) reference data, in which the DOM ground GSD geometric resolution was 0.2 m, and the plane accuracy ≤1 m; the DEM geometry ground resolution was 1 m GSD, accuracy ≤2 m (Figure 4).

**Figure 5:**
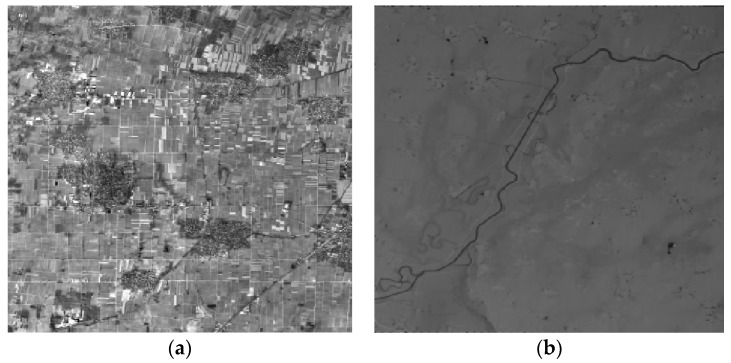
Reference data of the Anyang calibration field. (**a**) Digital orthophoto; (**b**) Digital elevation model.

The Anyang field is also located in Henan Province, China, featuring a plain, 114°19′–115°12′ E/35°44′–36°1′ N, coverage 90 × 30 = 2700 km^2^; average altitude approximately 40 m (the highest point is 70 m), and a maximum fluctuation ≤100 m. The field provides a region of 1:1000-scale DOM and DEM reference data, in which the DOM ground GSD geometric resolution was ≤0.1 m, and the plane accuracy ≤0.5 m; the DEM ground GSD geometric resolution was ≤0.5 m, accuracy ≤1 m (Figure 5).

### 3.2. Experimental Results and Analysis

(1) Quality of the star sensor observation data

Star sensors are connected through a bracket and are vertically fixed. In theory, the angle between any two optical axes of the star sensor should be a constant. Therefore, we did a quality analysis on the original observation data using variation detection means of the optical axis angles. The general auxiliary data included only raw observations of the Astro10A and Astro10B star sensors, we focused on the raw data of the two sensors for our analysis.

**Figure 6:**
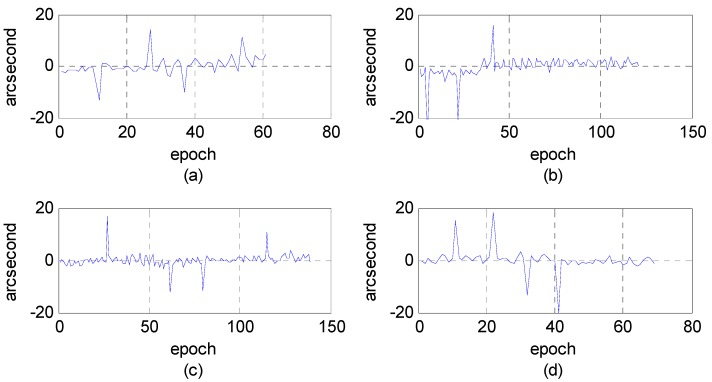
Variations of the star sensor optical axis angle before treatment using photos taken in different calibration fields. (**a**) Yili field; (**b**) Songshan field; (**c**,**d**) Anyang field.

**Figure 7:**
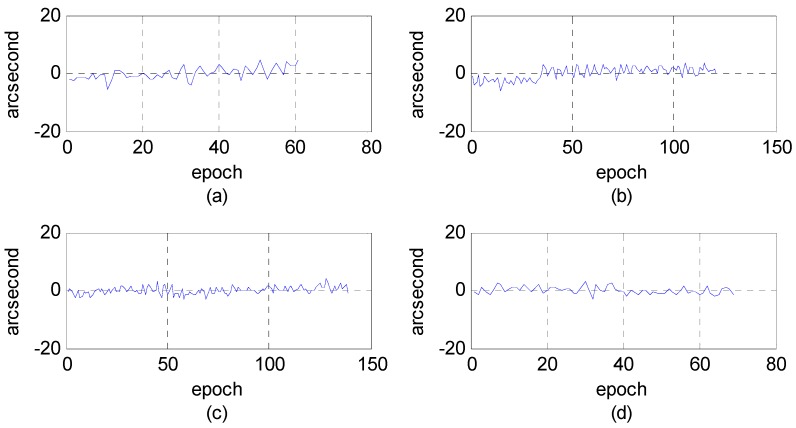
Variations of the star sensor optical axis angle after treatment using photos taken in different calibration fields. (**a**) Yili field; (**b**) Songshan field; (**c**,**d**) Anyang field.

First, we analyzed the quality of the raw observations in the angle changes of the star sensors. Figure 6 and Figure 7 show the errors in the angle change before and after treatment. Because the measurement accuracy of the optical axis of Astro10 star sensor is ≤5 arcsec, according to the law of error propagation, the optical axis angle error of the star sensor would be ≤7 arcsec.

As Figure 6 depicts, when a photo was taken in the Yili, Anyang, or Songshan calibration field, gross errors of 20–40 arcsec were presented in the observation data during some epochs that are much greater than the star sensor measurement accuracy. After being preprocessed in our algorithm, the gross errors were effectively corrected, and the optical axis angle error could satisfy the indicator (Figure 7).

**Figure 8:**
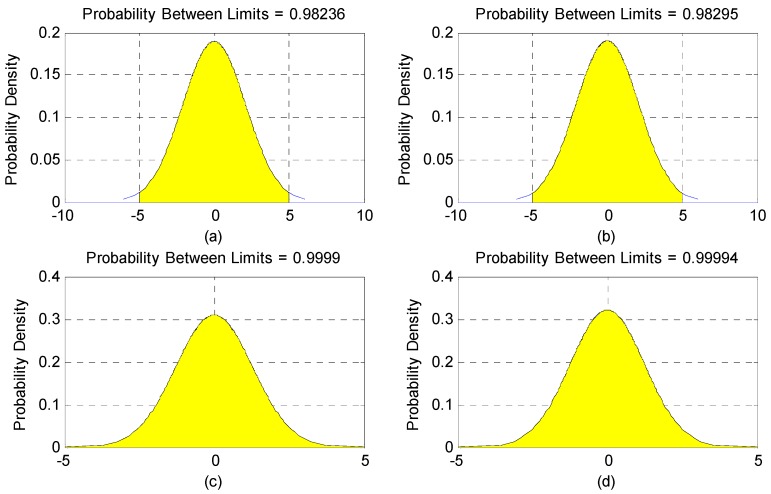
Normal distribution of the variations in star sensor optical axis angle when photos were taken in different calibration fields. (**a**) Yili field; (**b**) Songshan field; (**c**,**d**) Anyang field.

Figure 8 represents the distribution of the variations in star sensor optical axis angle error, Table 2 represents the statistics of the optical axis angle error characteristic. As Figure 8 and Table 2 show, the error of the angle change followed a normal distribution. However, the gross errors in the observations did not exist, and the chance of a ±5 arcsec deviation appearing between the optical axes was 95%.

**Figure 9:**
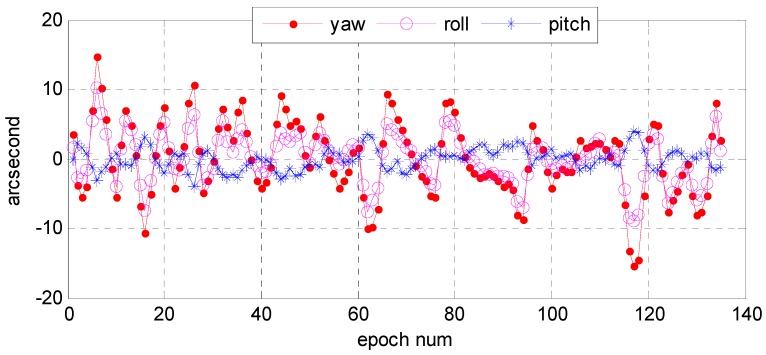
The original observation accuracies of the Astro10A star sensor when photos were taken in the Anyang calibration field on 1 January 2015.

**Figure 10:**
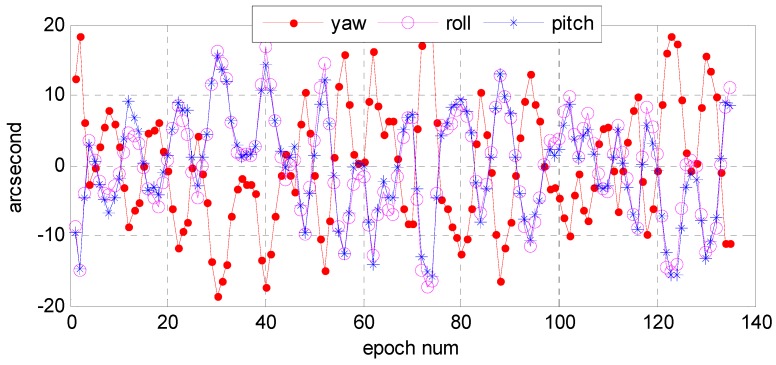
The original observation accuracies of the Astro10B star sensor when photos were taken in the Anyang calibration field on 1 January 2015.

**Figure 11:**
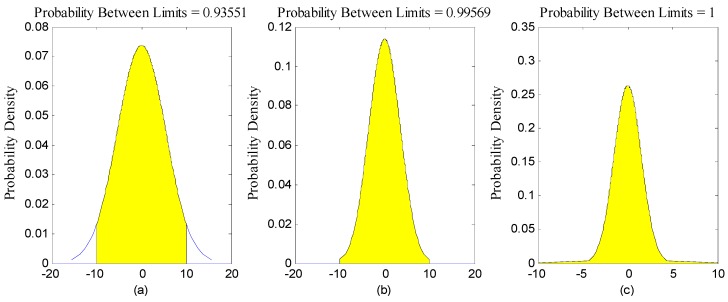
Normal distribution of the original observation accuracies of the Astro10A star sensor (**a**) yaw; (**b**) roll; (**c**) pitch.

**Figure 12:**
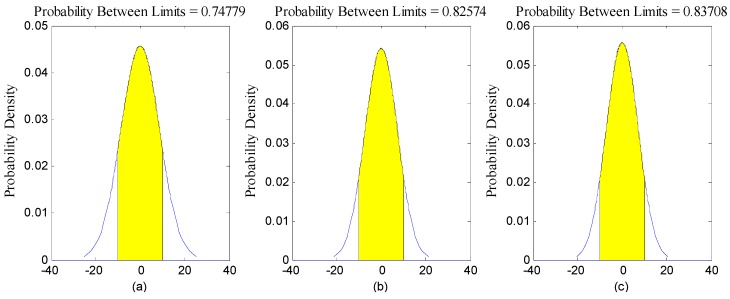
Normal distribution of the original observation accuracies of the Astro10B star sensor (**a**) yaw; (**b**) roll; (**c**) pitch.

**Figure 13:**
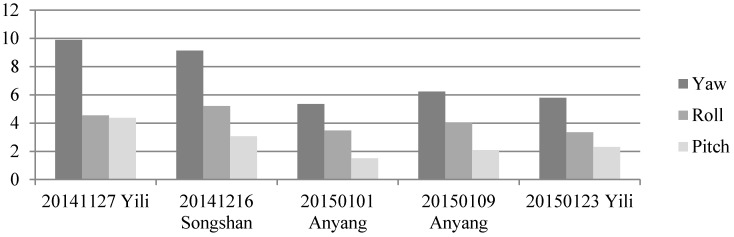
Precision of the onboard Astro10A original observation data at different times and places (unit: arcsec).

**Figure 14:**
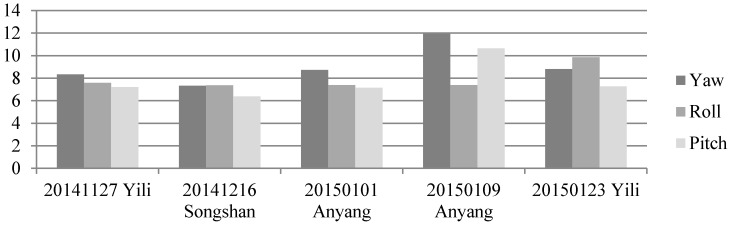
Precision of the onboard Astro10B original observation data at different times and places (unit: arcsec).

As described in Section 2.6, we would further use those precise inversion attitude parameters as reference data to analyze the relative precision of the star sensor raw observation data. Figure 9 and Figure 10 show, respectively, the error distributions in the Astro10A and Astro10B star sensors in the yaw, roll, and pitch directions. The maximum deviations in arcsec were −15–15 in yaw direction, −10–10 in roll direction, and −5–5 in pitch direction, while those of Astro10B were −15–20, −15–15, and −15–15 arcsec, respectively. This was because only the optical axis of star sensor could achieve the high pointing accuracy, the single star sensor attitude determination accuracy was limited and could not be directly used for attitude determination. In addition, the error distribution in the three directions of the two sensors were all normal in distribution (Figure 11 and Figure 12); and the relative precision of the measurements in the three directions were ≤12 arcsec (Figure 13 and Figure 14), which was consistent with the star sensor design accuracy of the optical axis error was ≤5″ (3σ) and horizontal axis error was ≤35″ (3σ). Therefore, the data quality of the star sensor observation used in our experiment will be reliable for other processes.

(2) Convergence and stability after bidirectional filtering

On the basis of the measurement and state equations, we used a bidirectional filter and overall weighted smoothing method to process the attitude data. Raw observational attitude data taken in the Anyang calibration field on 1 January 2015 were applied for data fusion, and the experimental results were analyzed.

**Figure 15:**
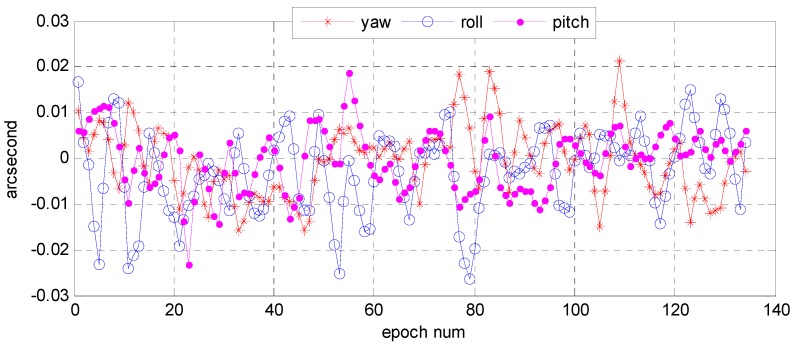
Variation of Euler angle error based on star sensor and gyro information fusion.

**Figure 16:**
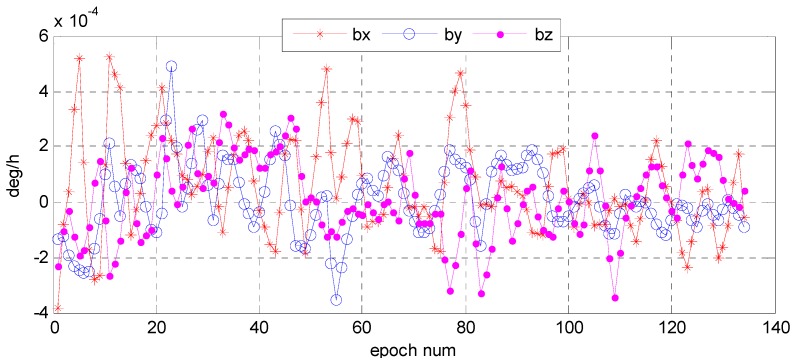
Variation of error bias based on star sensor and gyro information fusion.

**Figure 17:**
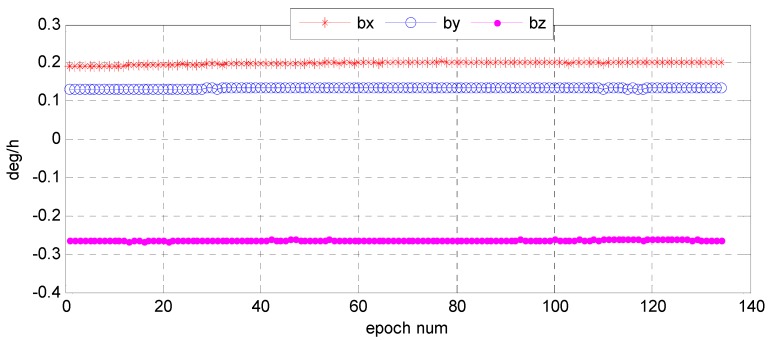
Gyro bias estimates after star sensor and gyro information fusion.

Understanding the variation trend of a state error parameter is important to determine whether the filter for the attitude determination system is convergent and stable. To test the convergence and stability of bidirectional filter and overall weighted smoothing method, we had chosen state error characteristic parameters of three vector parameter variables of attitude error quaternion, $X={\left[\Delta q_{13}{}^{T},\Delta b^{T}\right]}^{T}$, $\Delta q_{13}={\left[\Delta q_{1}\;\;\;\Delta q_{2}\;\;\;\Delta q_{3}\right]}^{T}$, and the gyro bias error as state variables of the system. The error quaternion means the difference between the predicted and the corrected respectively by gyro and star sensor, and the error of the gyro bias means that was corrected by the star sensor. With the bidirectional filter on the star sensor and the gyro, information can be fused and the variation trend of the error parameters can stand out (Figure 15 and Figure 16). We found that Euler angle errors in the yaw, roll, and pitch directions varied steadily and randomly, and so did the gyro. The range of the Euler angle error was −0.02–0.03 arcsec, and that of error bias −4.0 × 10^−4^~6.0 × 10^−4^ deg/h. The estimates for gyro bias changed over time (Figure 17). Therefore, we conclude that the gyro bias in the *X*, *Y*, and *Z* directions tended toward a constant value within 0.2 deg/h, meeting the gyro bias requirement of ≤2.0 deg/h.

**Figure 18:**
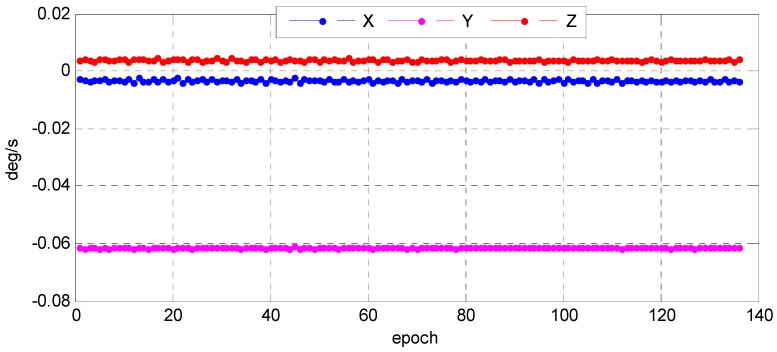
Gyro angle velocity estimates after star sensor and gyro information fusion.

In order to further verify the convergence and stability of the designed filter, we present a detailed description of the change in the gyro angle velocity estimates after star sensor and gyro information fusion. As shown in Figure 18, the values of the gyro angle velocity in three directions became close to the true state of the satellite flight that when the satellite was in stable flight, the angular velocity measured by the gyro was the angular velocity of the satellite around the Earth, about 0.06 degrees per second, and the measurement noise was effectively smoothed out. For more details, we list the average value and mean square of the Euler angle and error bias errors in the photos taken in different calibration fields at different times, and the two errors tended toward a Gaussian distribution (Table 3 and Table 4). Therefore, the bidirectional Kalman filter was reliable, which could maintain the convergence and stability.

(3) Relative attitude accuracy

**Figure 19:**
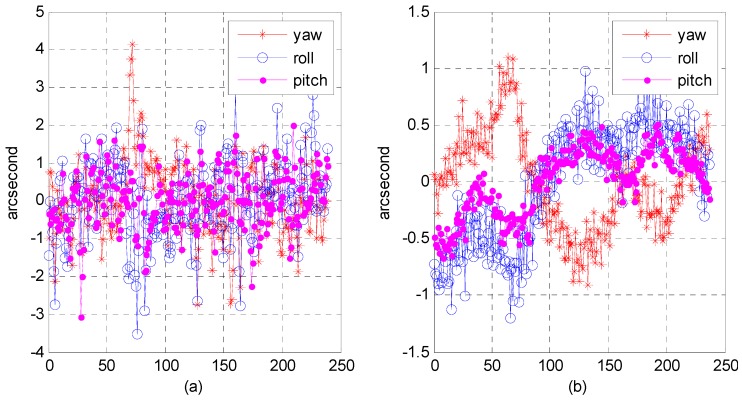
Distribution of relative attitude error using photos taken in the Yili calibration field: (**a**) in multi-star sensor combination; (**b**) in star sensor and gyro combination.

**Figure 20:**
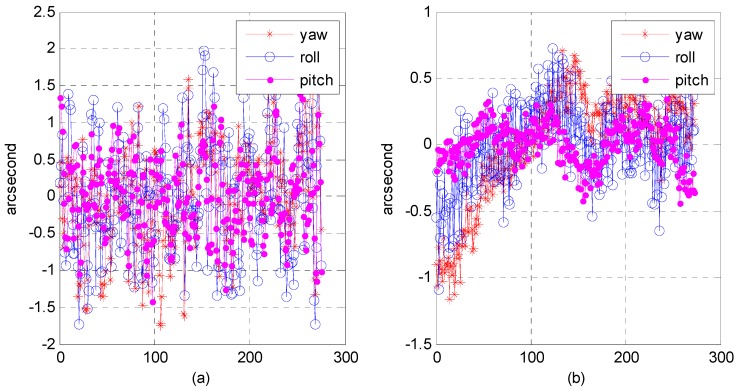
Distribution of relative attitude error using photos taken in the Anyang calibration field: (**a**) in multi-star sensor combination; (**b**) in star sensor and gyro combination.

**Figure 21:**
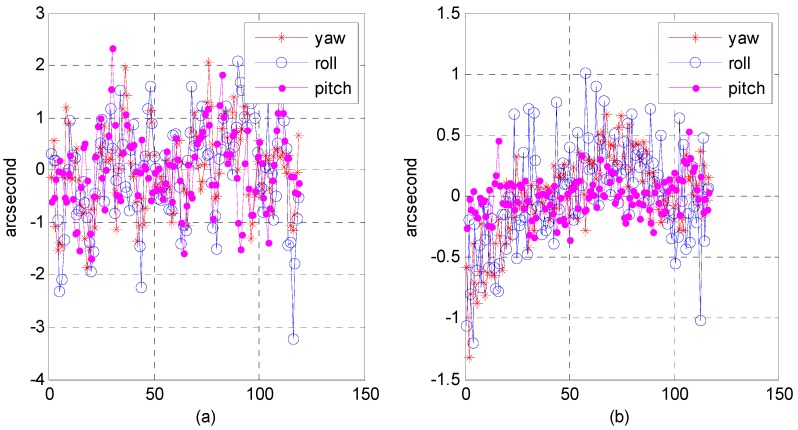
Distribution of relative attitude error using photos taken in the Songshan calibration field: (**a**) in multi-star sensor combination; (**b**) in star sensor and gyro combination.

Due to the nature of attitude data, it is difficult to verify their accuracy and reliability. As described in Section 2.6, we still used precise attitude data calculated from an optical image in the geometric calibration field as reference data. We converted attitude parameters from the body coordinate system relative to the inertial coordinate into the body coordinate relative to the orbit coordinate. It is more convenient for us to analyze the processing precision of optical image geometry in the along-orbit direction and the direction perpendicular to the orbit. Figure 19, Figure 20 and Figure 21 present the relative attitude error distribution using photos taken in the Yili, Anyang, and Songshan calibration fields. As figure parts (a) and (b) indicate, we used a multi-star sensor combination and a star sensor and gyro combination to process the attitude data.

The results show that in the multi-star sensor combination, the range of relative attitude error in the yaw, roll, and pitch directions could reach the level of −2–3 arcsec, while in the star sensor and gyro combination, the range of relative attitude error could be within plus or minus sub-arcsec level. We analyzed the reliability of our proposed algorithm; Table 5 presents the statistics on the mean square error of the relative attitude in different attitude-sensor combinations. In the case of the multi-star sensor combination, the mean square error could reach approximately 1 arcsec and was ≤0.5 arcsec in the star sensor and gyro combination. The processing effect in the multi-star sensor combination was worse than in the star sensor and gyro combination because the attitude in the multi-star sensor combination contained high-frequency noise; in the star sensor and gyro combination, star sensor high-frequency noise could be smoothed out, and the gyro bias could be estimated to optimize the attitude data estimation.

(4) Accuracy analysis of attitude fitting model

Although the precision attitude parameters could be obtained through a star sensor and gyro combination, the sampling frequency was only 4–8 Hz, far below the imaging frequency (20,000 Hz) of the satellite line push-broom camera. The resolution of the satellite panchromatic camera in this research was 1 m and the orbital altitude was 645 km; to meet a panchromatic geometric relative accuracy of better than 1 pixel, the fitting model accuracy of the attitude parameter should be within 0.3 arcsec. Therefore, we focused on how to obtain the precise attitude parameters of each scan line of the push-broom camera. Three attitude-fitting methods are described in detail in Section 2.5: Lagrange polynomial interpolation, orthogonal polynomial fitting, and the spherical linear interpolation model. We used different fitting models in our experiment on attitude parameters obtained from the star sensor and gyro combination, and evaluated the fitting accuracy in its precise attitude.

**Figure 22:**
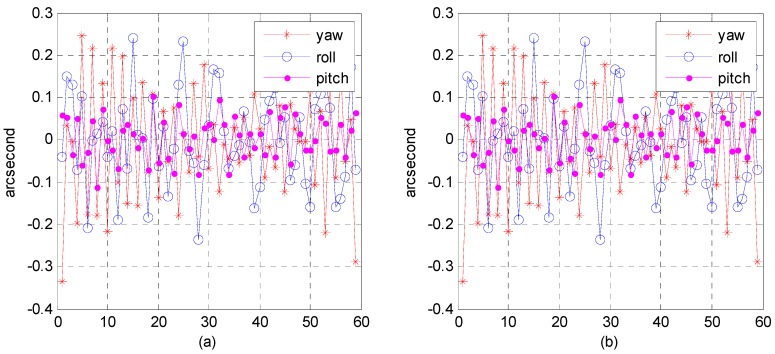
Distribution of attitude fitting accuracy in the Lagrange polynomial interpolation model: (**a**) fitting based on Euler angle parameters; (**b**) fitting based on quaternion parameters.

**Figure 23:**
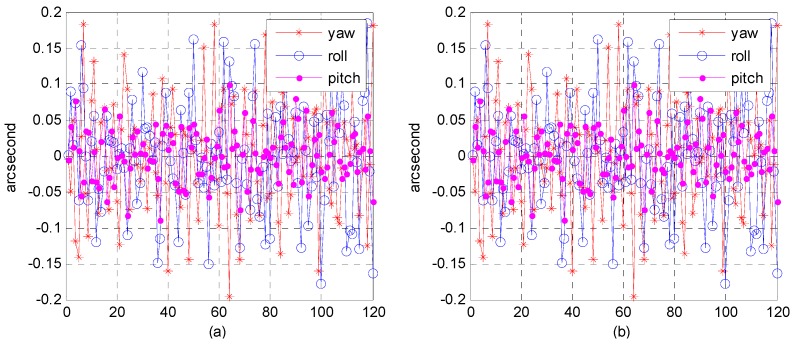
Distribution of attitude-fitting accuracy in the orthogonal polynomial fitting model: (**a**) Fitting based on Euler angle parameters; (**b**) Fitting based on quaternion parameters.

**Figure 24:**
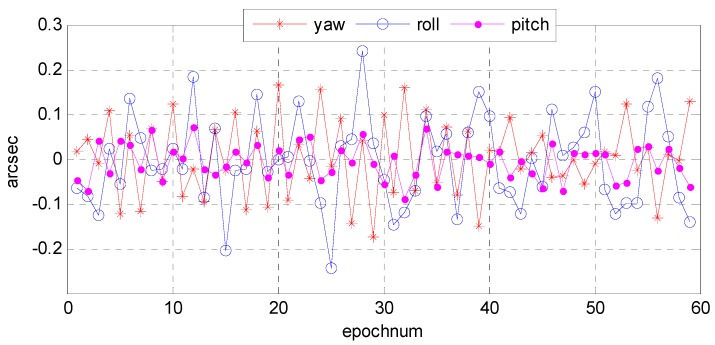
Distribution of attitude fitting accuracy in the spherical linear interpolation model.

The attitude data used to fit on different fitting models were down-sampled from 8 to 4 Hz, allowing us to use different fitting models to fit attitude parameters of different forms of expression. Finally, we used 8 Hz attitude parameters as reference values to evaluate the fitting accuracy. Figure 22, Figure 23 and Figure 24, describe respectively, the attitude fitting accuracy distribution in the Lagrange polynomial interpolation, orthogonal polynomial fitting, and spherical linear interpolation models.

Parts (a) and (b) in each figure separately indicate the attitude parameter in the Euler angle and quaternion. The above-mentioned figures lead us to believe that we can control the attitude fitting accuracy in yaw, roll, and pitch directions within a level of 0.3 arcsec. Attitude parameters of both the Euler angle and quaternion could be used to build mathematical fitting models, and all of three types of fitting model could be used to fit the attitude parameter. Table 6 lists the statistical details of the fitting accuracy in the yaw, roll, and pitch directions based on different fitting methods. The results show that the orthogonal polynomial fitting model was more suitable for building mathematical models and could ensure the relative geometry accuracy of the optical image.

(5) Imagery processing accuracy

We would use post-precise orbit data to provide precise exterior orientation line elements for image geometry processing, in which the accuracy could reach the centimeter level. Therefore, due to high-precision calibration and time synchronization, the attitude data are used directly for image geometry processing, which may directly reflect the quality of the data. The Section 3.1 describes in detail the geometric calibration field data for checking geometric accuracy, including the Songshan, Anyang, Dongying, Sanya, Taiyuan, and Yili calibration fields, and we would check the quality of the attitude parameter on the basis of the geometric calibration field image taken by the panchromatic camera and DOM/DEM reference data.

**Figure 25:**
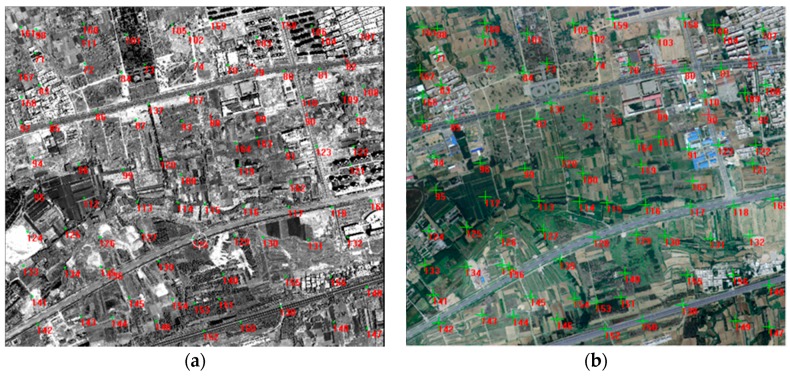
Correspondence points between the DOM image and geometric correction image of the satellite panchromatic camera taken in Songshan on 16 March 2015: (**a**) panchromatic image; (**b**) reference image.

**Figure 26:**
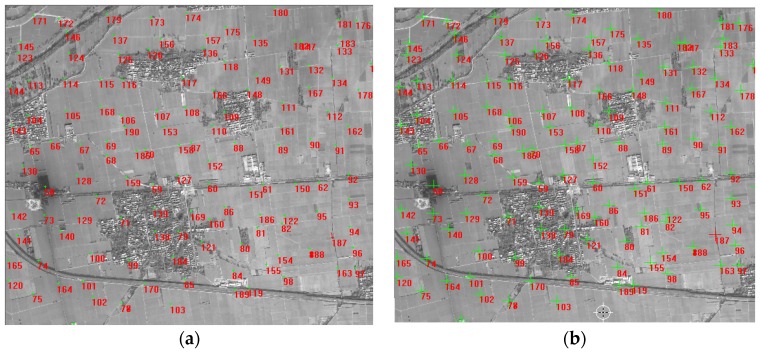
Correspondence points between the DOM and the geometric correction images of the satellite panchromatic camera taken in Anyang on 9 February 2015: (**a**) panchromatic image; (**b**) reference image.

**Figure 27:**
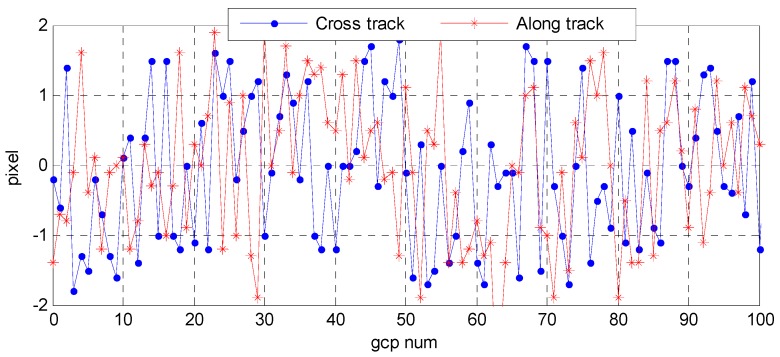
Distribution of the geometric relative accuracy in the cross-track and along-track directions of a panchromatic image taken in Songshan on 16 March 2015.

**Figure 28:**
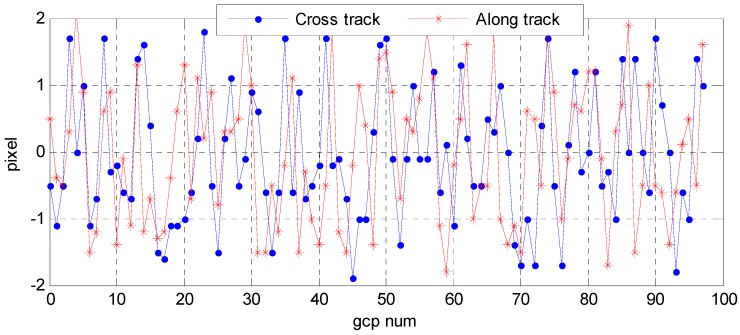
Distribution of the geometric relative accuracy in the cross-track and along-track directions of a panchromatic image taken in Anyang on 9 February 2015.

Wuhan University developed an optical satellite ground pretreatment system for the Yaogan-24 remote sensing satellite image processing, and we conducted experiments on the platform. The optical satellite ground pretreatment includes radiation treatment, sensor calibration, geometric correction, and so on. We took attitude data as the input for image preprocessing, and then analyzed the geometric accuracy on the basis of the geometric correction product and DOM/DEM of the calibration field reference data. Figure 25 and Figure 26 show the distribution of correspondence points between the DOM images and geometric correction images taken by the satellite panchromatic camera in Songshan on 16 March 2015 and in Anyang on 9 February 2015. The correspondence points between the DOM and geometric correction images were sufficient and were distributed more evenly to ensure the reliability of the geometric precision. Figure 27 and Figure 28 show the distribution of the relative geometric accuracy in the cross-track and along-track directions of the panchromatic image with respect to the DOM/DEM of the calibration field. The relative geometric accuracies in the cross-track and along-track directions were clearly between 1.5 and 2.0 pixels. Meanwhile, we concluded that the relative accuracy in the attitude data was 0.3–0.5 arcsec with respect to the precise attitude data calculated on the optical image in the geometric calibration field in Section 3.2. Therefore, both conclusions confirmed each other. Table 7 and Table 8 respectively show detailed statistics on the uncontrolled and relative positioning accuracy of the satellite geometric correction image taken by the panchromatic camera based on on-board and on-ground processing attitude data. Furthermore, we could conclude that the side swing angle did not affect the image geometric correction accuracy under no control-point condition when images were taken in different calibration fields, and the uncontrolled and relative positioning accuracy of the geometric correction image based on on-ground processing attitude data was about 15 m and 1.3 pixels, comparing to on-board attitude data about 30 m and 2.4 pixels, which increased about 50%. As the attitude determination accuracy of the star sensor was 5 arcsec, which is configured by the satellite, and the orbital altitude of the satellite was 645 km, 1 arcsec corresponded to a ground error of 3.127 m. Theoretically, the uncontrolled positioning precision of the satellite should within 15 m. Therefore, the attitude determination accuracy of the star sensor and the image positioning accuracy without a control point were consistent.

## 4. Conclusions

In this paper, we proposed a method of on-ground processing for attitude data of the Yaogan-24 remote sensing satellite and verification based on the geometric calibration field. By addressing the algorithms, we achieved a significant result. First, the quality of the star sensor observation data can be effectively preprocessed, and the optical axis angle error and original observations error in the directions of yaw, roll, and pitch were in line with the accuracy of the star sensor design indicators and were normally distributed. In addition, application of the bidirectional filter and overall weighted smoothing for attitude data information fusion and performance evaluation showed that the on-ground processing model could achieving bidirectional convergence, ensuring the robust and feasible. Furthermore, different attitude fitting models were analyzed. The results showed that both the attitude parameter of the Euler angle and quaternion could be used to build a mathematical fitting model, in which the orthogonal polynomial fitting model was more suitable for building mathematical models and ensured the relative geometry accuracy.

Finally, how to evaluate the relative and absolute accuracies of the attitude result obtained from the proposed algorithm was important. The experimental results show that the relative accuracy of the attitude data was 0.3–0.5 arcsec, and the relative geometric accuracy in the cross-track and along-track directions was between 1.5 and 2.0 pixels. The attitude determination accuracy of star sensor configured by the satellite was 5 arcsec, while the uncontrolled positioning precision of satellite’s panchromatic image was within 15 m. Therefore, both conclusions confirmed each other. In addition, the uncontrolled and relative geometric positioning accuracy of the panchromatic image could be effectively improved, when comparing to the result based on on-board attitude data.

Note that this paper does not involve topics such as error sources in the attitude sensor and sensor calibration. In the future, we will focus on the error characteristics, error model construction, relative and absolute calibration model construction, and the effect of the calibration parameters generated on the image geometric precision. Moreover, because the accuracy and frequency of the attitude data have become a key factor in high-resolution optical satellite image geometry processing, the attitude data of the high-frequency angular displacement sensor will be considered and discussed in future work.

## Figures and Tables

**Table 1:** The Yaogan-24 remote sensing satellite’s star sensor and gyro performance parameters.

Attitude Sensor	Performance Parameters
Star sensor ASTRO10	Optical axis error ≤5″ (3σ)
	Horizontal axis error ≤35″ (3σ)
	Frequency 4 Hz
APS star sensor	Optical axis error ≤20″ (3σ)
	Horizontal axis error ≤35″ (3σ)
	Frequency 4 Hz
Gyro components	Random bias ≤0.39°/h (3σ)
	Constant bias ≤2°/h
	Frequency 8 Hz

**Table 2:** Statistics of the optical axis angle change in the Astro10A and Astro10B star sensors in different times and places (unit: arcsec).

	Average	Skewness	Kurtosis	Mean Square Error
27 November 2014 Yili	2.3483 × 10^−11^	0.117	2.868	2.089
16 December 2014 Songshan	−1.3952 × 10^−11^	−0.438	2.360	2.087
24 December 2014 Anyang	−1.15568 × 10^−10^	0.188	3.213	1.282
1 January 2015 Anyang	5.1159 × 10^−11^	0.100	2.789	1.231
23 January 2015 Yili	7.548 × 10^−12^	−0.034	2.285	1.669

**Table 3:** Statistics of the Euler angle error at different times and places (unit: arcsec).

	Average Value			Mean Square Error		
Calibration Fields	Yaw	Roll	Pitch	Yaw	Roll	Pitch
Yili	0.00156	0.00035	0.00031	0.00863	0.01199	0.01067
Anyang	−0.00026	0.00003	0.00041	0.00423	0.00429	0.00333
Anyang	−0.00019	−0.00017	0.00018	0.01194	0.00991	0.01013
Yili	−0.00164	0.00300	0.00119	0.01045	0.01298	0.00988
Songshan	−0.00109	−0.00132	−0.00008	0.00717	0.00732	0.00542

**Table 4:** Statistics of gyro bias error at different times and places (unit: deg/h).

	Average Value			Mean Square Error		
Calibration Fields	B*_x_*	B*_y_*	B*_z_*	B*_x_*	B*_y_*	B*_z_*
Yili	−9.7333 × 10^−6^	−7.3 × 10^−6^	−3.5 × 10^−5^	0.00026	0.000237	0.000188
Anyang	−4.55149 × 10^−7^	−4 × 10^−6^	2.5 × 10^−6^	3.97 × 10^−5^	3.06 × 10^−5^	3.98 × 10^−5^
Anyang	4.19674 × 10^−6^	−4.5 × 10^−6^	4.98 × 10^−6^	0.000211	0.000218	0.000246
Yili	−6.8224 × 10^−5^	−2.7 × 10^−5^	3.84 × 10^−5^	0.000278	0.000214	0.000234
Songshan	1.52775 × 10^−5^	1.1 × 10^−6^	1.19 × 10^−5^	7.91 × 10^−5^	5.79 × 10^−5^	7.67 × 10^−5^

**Table 5:** Mean square error of the relative attitude in different attitude sensor combinations (unit: arcsec).

	Multi-Star Sensor CombInation			Star Sensor and Gyro Combination		
Calibration Field	Yaw	Roll	Pitch	Yaw	Roll	Pitch
Yili	0.727	0.868	0.811	0.272	0.611	0.477
Anyang	0.699	0.773	0.543	0.418	0.303	0.161
Anyang	1.005	0.782	0.796	0.326	0.386	0.294
Yili	0.789	0.993	0.733	0.354	0.416	0.159
Songshan	1.022	1.029	0.777	0.425	0.513	0.291
RMS	0.859	0.895	0.738	0.363	0.458	0.299

**Table 6:** The mean square error of attitude fitting accuracy in different fitting models (unit: arcsec).

	Fitting on Euler			Fitting on Quaternion		
Fitting Model	Yaw	Roll	Pitch	Yaw	Roll	Pitch
Lagrange polynomial	0.209	0.207	0.249	0.204	0.203	0.244
Orthogonal polynomial	0.141	0.101	0.136	0.142	0.105	0.135
Spherical linear	NULL	NULL	NULL	0.272	0.225	0.119

**Table 7:** Uncontrolled and relative positioning accuracy of a geometric correction image taken by the panchromatic camera based on on-board attitude data (unit: m).

Time	Side Swing (°)	Regions	Positioning Accuracy	Average Offset		Relative Accuracy	
				dx	dy	mx	my
27 December 2014 15:27	−3.820	Yili	19.643	16.788	−10.199	1.942	2.438
9 February 2015 11:10	−5.532	Anyang	25.364	21.329	13.727	2.101	1.854
16 March 2015 13:20	4.152	Songshan	31.570	−18.693	−25.441	2.478	2.135
1 May 2015 16:13	3.318	Anyang	10.883	9.233	−5.762	1.873	2.195
23 July 2015 15:27	−2.243	Yili	35.982	−34.288	10.912	2.711	2.389
12 September 2015 14:15	7.436	Dongying	29.998	22.788	−19.509	2.788	1.993
20 October 2015 13:30	−5.616	Sanya	45.548	37.255	−26.205	1.893	2.154
25 December 2015 16:55	2.378	Taiyuan	27.536	−24.406	−12.752	3.117	2.082
RMS			29.941	24.622	17.036	2.404	2.162

**Table 8:** Uncontrolled and relative positioning accuracy of a geometric correction image taken by the panchromatic camera based on ground processing attitude data (unit: m).

Time	Side Swing (°)	Regions	Positioning Accuracy	Average Offset		Relative Accuracy	
				dx	dy	mx	my
27 December 2014 15:27	−3.820	Yili	13.141	−11.581	−6.212	1.665	1.721
9 February 2015 11:10	−5.532	Anyang	6.429	5.356	−3.557	1.355	1.399
16 March 2015 13:20	4.152	Songshan	7.020	4.271	−5.572	1.323	1.413
1 May 2015 16:13	3.318	Anyang	8.381	−3.564	−7.586	0.831	1.896
23 July 2015 15:27	−2.243	Yili	15.747	−14.197	6.815	1.385	1.125
12 September 2015 14:15	7.436	Dongying	18.288	15.734	−9.323	1.255	1.133
20 October 2015 13:30	−5.616	Sanya	21.099	16.388	−13.29	1.421	0.847
25 December 2015 16:55	2.378	Taiyuan	17.676	−14.988	−9.372	1.006	1.155
RMS			14.464	11.916	8.197	1.302	1.374
